# Revolutionizing Localized Lung Cancer Treatment: Neoadjuvant Chemotherapy plus Immunotherapy for All?

**DOI:** 10.3390/jcm13092715

**Published:** 2024-05-05

**Authors:** Victoria Ferrari, Carole Helissey

**Affiliations:** 1 Centre Antoine Lacassagne, 06100 Nice, France; victoria.ferrari@nice.unicancer.fr; 2 Department of Medical Oncology and Clinical Research Unit, Military Hospital Bégin, 94160 Saint-Mandé, France

**Keywords:** non-small cell lung cancer (NSCLC), neoadjuvant therapy, chemotherapy, immunotherapy, personalized treatment

## Abstract

Lung cancer poses a significant public health challenge, with resectable non-small cell lung cancer (NSCLC) representing 20 to 25% of all NSCLC cases, staged between I and IIIA. Despite surgical interventions, patient survival remains unsatisfactory, with approximately 50% mortality within 5 years across early stages. While perioperative chemotherapy offers some benefit, outcomes vary. Therefore, novel therapeutic approaches are imperative to improve patient survival. The combination of chemotherapy and immunotherapy emerges as a promising avenue. In this review, we explore studies demonstrating the benefits of this combination therapy, its impact on surgical procedures, and patient quality of life. However, challenges persist, particularly for patients failing to achieve pathologic complete response (pCR), those with stage II lung cancer, and individuals with specific genetic mutations. Additionally, identifying predictive biomarkers remains challenging. Nevertheless, the integration of immunotherapy and chemotherapy in the preoperative setting presents a new paradigm in managing resectable lung cancer, heralding more effective and personalized treatments for patients.

## 1. Introduction

### 1.1. Overview and Epidemiology

Lung cancer represents a significant public health issue, with over 2,200,000 estimated new cases worldwide in 2020 [1]. Lung cancer exists in two main forms: Non-Small Cell Lung Cancer (NSCLC) which affects approximately 85% of patients, and Small Cell Lung Cancer (SCLC), which accounts for around 15% of cases [2]. Resectable NSCLC is defined by its staging between stage I and IIIA and accounts for approximately 20 to 25% of all stages of NSCLC [3].

If surgery remains the cornerstone of managing localized NSCLC, it can be supplemented with perioperative therapies depending on the extent of tumor involvement and locoregional lymph node status [4]. Despite these treatment combinations, the survival of patients with localized and surgically treated NSCLC remains unsatisfactory, with approximately 50% of patients deceased at 5 years, across all early stages [5].

### 1.2. Adjuvant and Neoadjuvant Chemotherapy

Several large trials have investigated the potential of adjuvant platinum-based doublet chemotherapy in resectable non-small cell lung cancer (NSCLC), with inconsistent confirmation of positive overall survival (OS) outcomes [6,7,8,9]. Ultimately, it was the LACE meta-analysis, which pooled the results of five randomized trials dating back to the 1995s on the use of adjuvant cisplatin, that confirmed the improvement in OS for surgically treated bronchial cancer patients, with an absolute benefit of 5% at 5 years. This established cisplatin as the standard chemotherapy in the postoperative setting [5].

Following the adoption of adjuvant cisplatin-based chemotherapy for NSCLC, several neoadjuvant studies closed due to accrual difficulties [10]. Although the primary interest of preoperative chemotherapy is to reduce tumor size to improve resectability, it also addresses potential micro-metastases not visible on imaging. In 2014, the NSCLC Collaborative Group pooled results from 15 randomized trials examining the benefits of cisplatin-based neoadjuvant chemotherapy in stage I to IIIA lung cancers, concluding a 5% improvement in patients’ 5-year OS [11]. 

However, Felip et al. assessed if adding preoperative or adjuvant chemotherapy to surgery extends disease-free survival in resectable non-small-cell lung cancer patients compared to surgery alone [12]. Of the 624 patients included, 97% of those randomized to the neoadjuvant chemotherapy arm received the full chemotherapy protocol, while only 66% of those randomized to the adjuvant chemotherapy arm received the intended chemotherapy regimen. Among early-stage patients, there were no statistically significant differences observed in disease-free survival when either preoperative or adjuvant chemotherapy was added to surgery [12].

Therefore, new therapeutic strategies need to be developed to enhance the survival of these patients.

## 2. Neoadjuvant Immunotherapy plus Chemotherapy: The State of Art

### 2.1. Rational for the Use of Immunotherapy in the Neoadjuvant Setting in Resectable Lung Cancer

With the emergence of immune checkpoint inhibitors (ICI) in metastatic lung cancers, following the arrival of initial positive studies in progression-free survival (PFS) and OS in monotherapy and subsequently in combination with chemotherapy [13,14,15,16,17,18,19,20,21] numerous studies have begun assessing the utility of immunotherapy in earlier settings.

The preclinical rationale for neoadjuvant immunotherapy is grounded in several concepts, including efficient antigen presentation by tumor cells to immune cells. This underscores the advantage of having a tumor in place, thus justifying the administration of immunotherapy prior to surgery. Indeed, this could bolster the response of anti-tumor T lymphocytes, given the presence of tumor burden and the subsequent increase in antigen presentation.

The additional benefits of immunotherapy used in the neoadjuvant setting are manifold: it allows for early treatment of micro-metastases while also contributing to tumor size reduction to enhance the feasibility of surgical resection, increase R0 resection rates and decrease the risks of postoperative complications. Moreover, it enables evaluation of the tumor stage of the surgical specimen: pathologic complete response (pCR) indicates the absence of viable tumor cells on the surgical specimen, while major pathologic response (MPR) signifies the presence of 10% or fewer viable tumor cells [22,23]. These two criteria are important as they could be correlated with disease-free survival and be considered surrogate endpoints for DFS. 

In a pilot study from 2018, the CheckMate 159, neoadjuvant administration of two cycles of nivolumab alone in 20 patients with localized stage I, II, and IIIA bronchial cancers was associated with a MPR in 45% of cases. Two of these patients achieved a pCR (10%) [24]. In the LCMC3 trial, a phase II study evaluating atezolizumab alone in the neoadjuvant setting for patients with stage IB–IIIB diseases (8th edition of the AJCC), two cycles of immunotherapy were administered prior to surgery [25]. Out of 181 patients, 159 underwent surgery within one and a half months after the first cycle of atezolizumab. Among the 143 evaluable patients, 6% achieved a pCR and 20% achieved a MPR (95% CI: 14–28%) on the surgical specimen (primary endpoint of the study). IONESCO, another phase II study, enrolled forty-six patients with resectable stage IB–IIIA non-N2 NSCLC. In this trial, three cycles of durvalumab alone were administered prior to surgery [26]. The primary endpoint was the percentage of complete surgical resection. Unfortunately, the study was prematurely terminated due to a high rate of 90-day postoperative mortality (9%).

Other trials have explored neoadjuvant immunotherapy either as monotherapy or in combination with a second immunotherapy agent, often an anti-CTLA4. This was the case in the NEOSTAR trial, which enrolled 44 patients with resectable NSCLC [27]. Of these, 23 received nivolumab monotherapy (three cycles prior to surgery), while 21 patients were randomized to the dual immunotherapy arm with nivolumab and ipilimumab, receiving three cycles of nivolumab and one cycle of ipilimumab concurrently with the first cycle of nivolumab. Over 90% of patients in both arms completed their preoperative treatment and underwent surgery. A MPR was achieved in 22% of patients in the nivolumab monotherapy arm compared to 38% in the nivolumab + ipilimumab combination arm.

Faced with promising yet still insufficient outcomes of neoadjuvant immunotherapy alone in localized lung cancers, new avenues have emerged, exploring the preoperative combination of chemotherapy and immunotherapy.

### 2.2. Combination of Chemo plus Immunotherapy before Surgery in Lung Cancer

We now recognize that the efficacy of chemotherapy extends beyond its cytotoxic effects, relying also on the immune system’s contribution, underscoring the crucial importance of immunocompetence in tumor combat. Several mechanisms may explain this synergy: chemotherapies promote tumor cell death, stimulating antigen presentation, while chemotherapy-induced lymphopenia can facilitate T cell expansion, thereby enhancing the immune response. Additionally, chemotherapy fosters the recruitment of specific immune cells within the tumor and prevents the suppressive function of certain immune cell populations, thus strengthening the antitumor immune response [22,28].

These findings highlight the potential of chemotherapy to boost the antitumor immune response by promoting antigen presentation and activating T cells within the tumor. It is therefore not surprising that the combination of chemotherapy with PD-1 and PD-L1-targeting immunotherapies has shown significant survival benefits, regardless of PD-L1 status, in metastatic non-small cell lung cancer.

NADIM, a single-arm multicenter phase II trial, focused on a patient population with more advanced disease among localized cancers [29]. This trial enrolled 46 patients with resectable stage IIIA non-small cell lung carcinoma (NSCLC), among whom 74% had N2 disease. Patients with stage I and II lung cancers were not included. The primary endpoint was 24-month progression-free survival (PFS), and patients received three cycles of neoadjuvant chemo-immunotherapy with Carboplatin, Paclitaxel, and Nivolumab. One year of Nivolumab was administered adjuvantly. The 24-month PFS was 77.1% (95% CI: 59.9–87.7). Pathological response was a key secondary endpoint, with 26 patients achieving a pCR (63%; 95% CI: 62–91) and 34 patients (83%, 95% CI: 68–93) achieving a MPR after surgery. Additionally, OS at 24 months was nearly 90% (95% CI: 74.5–96.2).

In a recent update, a 3-year progression-free survival of 69.6% (95% CI: 54.1–80.7) and an OS of 81.9% were observed in the intention-to-treat population. These results are significantly better than the survival data observed with neoadjuvant treatment in resectable NSCLC [24,25,27,30]. Thus, the NADIM study contributes to confirming the long-term efficacy of neoadjuvant chemoimmunotherapy in patients with resectable stage IIIA NSCLC.

In the continuum of NADIM and still focusing on patients with resectable stage IIIA NSCLC, NADIM II emerged as the first randomized phase II study in this context [31]. This time, 87 patients were enrolled and randomized to receive either neoadjuvant chemoimmunotherapy with carboplatin, paclitaxel, and nivolumab for three cycles, or neoadjuvant chemotherapy with carboplatin and paclitaxel for three cycles. Additionally, adjuvant treatment with Nivolumab was administered to patients achieving complete resection after surgery. The primary endpoint was the rate of pCR in the intention-to-treat population. Secondary endpoints included MPR, objective response rate (ORR), toxicity, and potential predictive biomarkers. In the chemoimmunotherapy group, the rate of pCR was significantly increased: 36.2% compared to 6.8% in patients without immunotherapy (99% CI: 1.32–20.87, *p* = 0.0071). Similar to the pCR, the MPR was also significantly better in patients receiving Nivolumab in addition to chemotherapy (52% vs. 14%). However, this study showed higher rates of grade 3 and 4 toxicities with nivolumab (24% vs. 10%) compared to chemotherapy alone. Once again, this study highlights the benefit of combining immunotherapy with neoadjuvant chemotherapy for operable locally advanced lung cancers, leading to superior histological responses. 

The CheckMate 816 trial was the first randomized controlled phase III trial to compare platinum-based neoadjuvant chemotherapy in combination with an ICI (nivolumab) vs. chemotherapy alone [32]. The dual primary endpoints were event-free survival (EFS) and pCR. Out of 358 enrolled patients, approximately 60% had stage IIIA disease. The median EFS was 31.6 months in the experimental arm (95% CI: 30.2 to not reached) and 20.8 months in the control arm (95% CI: 14.0–26.7). The risk of events was reduced by 37% in patients receiving the chemotherapy plus immunotherapy combination: HR of 0.63 (97.38% CI: 0.43–0.91; *p* = 0.005). Subgroup analyses showed better response rates in patients expressing PD-L1 ≥ 1% and in those with stage IIIA compared to stages I and II. Regarding pCR, it was 24% in the chemotherapy plus immunotherapy group vs. 2% in the chemotherapy alone group (OR of 13.94; 99% CI: 3.49–55.785; *p* < 0.001), and remained consistent across subgroups. 

Following the momentum created by CheckMate 816, several important studies continued to demonstrate the interest of a neoadjuvant chemotherapy plus immunotherapy combination in localized NSCLC. Among them, AEGEAN, KEYNOTE 671, CheckMate 77T, and finally the NEOTORCH study have garnered significant attention [33,34]. These studies also assessed the continuation of immunotherapy in the adjuvant setting. 

In the AEGAN phase III trial, Durvalumab is assessed in the perioperative setting, specifically in combination with platinum-based chemotherapy for 4 cycles in the experimental arm, compared to chemotherapy alone in the standard arm [33]. The anti-PD-1 treatment is continued adjuvantly for 12 months in the experimental arm vs. placebo in the standard arm. A total of 802 patients were included, of which approximately 70% had stage III disease (primarily IIIA). It is noteworthy that stage IIIB N2 patients were not excluded from the trial. The study’s two primary endpoints were EFS and pCR.

Overall, approximately 77% of patients in each arm underwent surgery, with a slightly higher proportion in the Durvalumab arm achieving R0 resection (94.7% vs. 91.3%). At 12 and 24 months, respectively, EFS was 73.4% in the chemotherapy plus immunotherapy arm (95% CI, 67.9 to 78.1) and 64.5% in the chemotherapy-only group (95% CI, 58.8 to 69.6), and 63.3% vs. 52.4% still in favor of adding immunotherapy (95% CI, 56.1 to 69.6 and 95% CI, 45.4 to 59.0). Pathological complete response was significantly better in patients who received immunotherapy with chemotherapy, with 17.2% vs. 4.3% achieving pCR in the standard arm.

Using a similar design but with pembrolizumab, the double-blind randomized phase III study KEYNOTE 671 also compared a combination of neoadjuvant chemotherapy plus immunotherapy (four cycles of platinum-based therapy) followed by adjuvant pembrolizumab for 13 cycles, to neoadjuvant chemotherapy plus placebo followed, post-surgery, by 13 cycles of placebo treatment [34]. Event-free survival and OSl were again the primary outcome measures. In this study, 797 patients with localized NSCLC stage II to IIIB (including N2 patients) were included. In the experimental arm, 82.1% of patients underwent surgery compared to 79.4% in the non-immunotherapy arm. The median EFS was not reached in the pembrolizumab group and was 17 months in the placebo group (95% CI; unreached to 34.1 months and 95% CI; 14.3 to 22, respectively). Recently, at ESMO 2023, unpublished OS results were presented, once again showing significantly better outcomes in patients who received immunotherapy with a median OS not reached vs. 45.5 months in patients with chemotherapy plus placebo (HR = 0.73 with 95% CI; 0.54 to 0.99. *p* = 0.02124). The KEYNOTE 671 study is the first trial combining neoadjuvant chemotherapy and immunotherapy plus adjuvant immunotherapy to demonstrate a significant benefit in OS.

CheckMate 77T (CM77T) a multicenter randomized double-blind phase III trial, is the first perioperative study to support the role of Nivolumab in the neoadjuvant setting, which has already been validated in practice through the CheckMate 816 trial [32,35].

In this trial, 461 patients with resectable stage IIA to IIIB non-small cell lung cancer (NSCLC) were randomized to receive either a combination of Nivolumab 360 mg every 3 weeks + platinum-based chemotherapy four cycles every 3 weeks followed by surgery and adjuvant Nivolumab for 12 months (480 mg/4 weeks), or placebo + chemotherapy before surgery followed by placebo adjuvant therapy for 12 months, on the same schedule as in the experimental arm. Event-free survival was the primary endpoint: at 12 months it was 73% vs. 59% in the experimental and control arms respectively. The median EFS was not reached in the Nivolumab arm, and was 18.4 months in the placebo arm. The addition of Nivolumab immunotherapy in this study showed a 42% reduction in the risk of events in favor of the experimental arm (*p* = 0.00025). At 18 months, the superiority of the combination with immunotherapy is still evident with an EFS of 70% vs. 50% with chemotherapy + placebo.

Among the exploratory secondary endpoints analyzed in the CM77T, pCR is an additional argument in favor of the superiority of the experimental arm as it is 25.3% with immunotherapy compared to 4.7% with placebo. Similarly, the rate of MPR is significantly higher at 35.4% vs. 12.1%. To date, OS has not yet been determined but everything suggests that it will also be significantly higher in the perioperative immunotherapy + chemotherapy arm, mirroring the results of the KEYNOTE 671 [34].

Since ASCO 2023, the NEOTORCH study has attracted considerable interest. This Chinese randomized double-blind, placebo-controlled phase III trial investigated the efficacy and safety of toripalimab, a novel PD1 inhibitor, in the perioperative setting for resectable stage II and III non-squamous bronchial carcinomas [36].

In the experimental group, toripalimab was combined with a platinum-based chemotherapy for 3 cycles in the neoadjuvant setting, followed by adjuvant administration for approximately one year, while patients in the standard group received platinum-based chemotherapy alone. A total of 501 patients were included, of whom 404 had stage III non-squamous bronchial carcinoma. The 97 patients with stage II were excluded from the interim analysis. The primary endpoints were EFS and MPR.

In the toripalimab group, EFS did not reach its median, whereas it was 15.1 months in the standard group. A significant reduction in the risk of events in favor of the chemotherapy + anti-PD1 combination was observed, with a decrease of 60% (HR = 0.40, 95% CI 0.277 to 0.565, and *p* < 0.0001). Additionally, the MPR was also better in the toripalimab group, with 48.5% pCR vs. 8.4%, and 24.8% pCR vs. 1%. As for OS, a secondary endpoint, it has not yet been determined, but interim analyses already seem to indicate a favorable profile for toripalimab.

The results of 43 trials (including 8 randomized trials) involving more than 5400 patients who received neoadjuvant chemoimmunotherapy for NSCLC were summarized in a meta-analysis published in early 2024 [37].

Pooling overall survival results from randomized controlled trials, this meta-analysis found improved overall survival in patients treated with neoadjuvant chemoimmunotherapy (HR, 0.65; 95% CI, 0.54–0.79; I2 = 0%). Similarly, patients who appeared to benefit most from the combination were those with PD-L1-positive status (≥1%) (HR, 0.49; 95% CI, 0.33–0.73; I2 = 48.5%) and those with stage III cancer (HR, 0.67; 95% CI, 0.53–0.85; I2 = 0%). EFS was also higher when patients received neoadjuvant chemoimmunotherapy compared to neoadjuvant chemotherapy (HR, 0.59; 95% CI, 0.52–0.67; I2 = 14.9%).

Regarding surgery, neoadjuvant chemoimmunotherapy vs. neoadjuvant chemotherapy alone was associated with higher rates of pathologic response (complete or major). Patients were more likely to undergo surgery and complete resection (R0), and the number of lobectomies was greater when immunotherapy was combined with chemotherapy. 

Finally, no significant difference in adverse events was observed between neoadjuvant immunotherapy and neoadjuvant chemotherapy in this meta-analysis.

Table 1 summarize all trials and their effectiveness.

**Figure 1:**
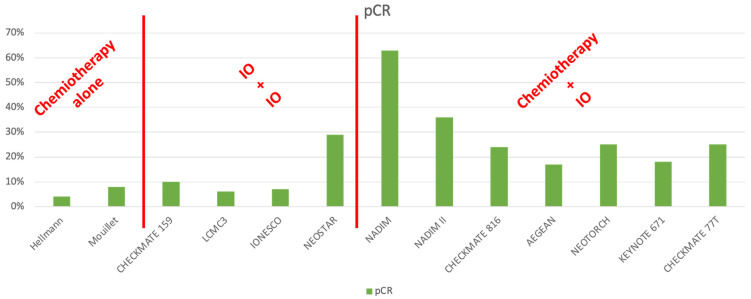
Rate of pCR according to perioperative treatments. IO: Immunotherapy.

The Figure 1 summarizes the response rate according to the perioperative treatment protocol.

### 2.3. Safety and PROs (QoL)

On average, according to studies, it is estimated that 15 to 20% of patients did not undergo surgery after their neoadjuvant treatment, with data still favoring immunotherapy. In less than 10% of cases, disease progression was the reason. The addition of chemotherapy combined with immunotherapy, besides being effective, did not lead to any difficulty or delay in surgery. Thus, 83.2% of patients in the chemotherapy plus Nivolumab arm were able to undergo surgery in the CheckMate 816 study, 77.6% of patients under chemotherapy plus durvalumab in the AEGEAN study, and finally 78% in the chemotherapy plus Nivolumab arm again in the CheckMate 77T [32,33,35].

On average, the percentage of patients with complete resection (R0) was around 90% and was higher in the arm with the addition of immunotherapy: 92% of patients under chemotherapy plus pembrolizumab in the KEYNOTE 671, 94.7% in the AEGEAN study with durvalumab, and 90% with nivolumab in the CheckMate 77T. This figure was slightly lower in the CheckMate 816 study with 83.2% complete resection but still remained higher than the arm without immunotherapy (77.8%) [32,33,34,35].

In this study, there were no additional difficulties shown in surgery with the addition of nivolumab. On the contrary, operation times seemed to be improved in the arm with immunotherapy, and surgeries were less invasive than in the control arm (fewer pneumonectomies and more minimally invasive surgeries).

However, it is worth noting that, according to studies, it is estimated that 15 to 20% of patients did not undergo surgery after their neoadjuvant treatment, with data still favoring immunotherapy. In less than 10% of cases, disease progression was the reason. Combining chemotherapy with immunotherapy may help identify and select rapidly progressing patients for whom surgery would not provide any benefit. This hypothesis needs to be validated in this population ineligible for surgery.

Regarding adverse effects under chemotherapy plus immunotherapy, no worrying deviations were highlighted. In CheckMate 816, more adverse effects were found under chemotherapy alone than when adding immunotherapy (97.2% vs. 92.6%), including considering only grades 3 and 4 (36.9% under chemotherapy alone vs. 33.5% with the combination). Adding durvalumab in the AEGEAN study also did not result in excess severe adverse effects (32.4% vs. 32.9% with and without immunotherapy, respectively) [32,33].

KEYNOTE 671 and CheckMate 77T showed that the combination of chemotherapy plus immunotherapy was responsible for a slight increase in adverse effects, with 44.9% of grade 3 or higher adverse effects under chemotherapy plus pembrolizumab compared to 37.3% under placebo, and 34% vs. 27% in CheckMate 77T [34,35].

Table 2 summarizes the types of surgery following neoadjuvant chemotherapy and immunotherapy, along with their associated safety.

Published more recently, the results of the Neo-Pre-Ic study, a Chinese single-arm phase II trial, tend to confirm the efficacy and safety of neoadjuvant immunotherapy by combining sintilimab with chemotherapy in patients with stage IIIA and B non-small cell lung cancer (NSCLC) deemed ineligible for upfront R0 surgery [38]. In this study thirty patients received a combination of sintilimab plus carboplatin and nab-paclitaxel for 2 to 3 cycles, followed by surgery, which was completed for 20 of them (66.6%). 

While this study confirms the efficacy of immunotherapy added to neoadjuvant chemotherapy, with pathological complete response (pCR) and major pathological response (MPR) rates reaching 40% and 75% respectively, and disease-free survival evaluated at 75% at 2 years, it also provides further arguments in favor of the safe use of this therapeutic combination.

Indeed, treatment-related adverse events were observed in 73% of patients, with half being grade I or II. Three patients experienced grade 3 or higher immune-related adverse events (irAEs). No dose reduction, treatment interruption, surgical delay, or deaths were observed during neoadjuvant treatment.

## 3. Challenges

The integration of chemotherapy and immunotherapy in the neoadjuvant setting has revolutionized the management of localized non-small cell lung cancer (NSCLC), leading to a significant increase in complete pathological response (pCR) rates observed across various clinical trials. However, pivotal questions persist. These include concerns about patients who do not achieve a pCR after receiving the combined chemotherapy and immunotherapy regimen, the application of this approach in patients with specific genetic mutations such as EGFR or ALK alterations, and its efficacy in patients with stage II disease.

Furthermore, questions arise regarding the role of maintenance therapy following either a pCR or non-pCR. Should the same molecular agents be maintained? Additionally, what is the role of adjuvant therapy in this context? 

### 3.1. Patients with Non pCR

As discussed, achieving complete pathological response (pCR) is a marker strongly associated with survival [39,40]. In the era of neoadjuvant chemotherapy alone, nearly 96% of patients did not achieve pCR [41,42]. The 5-year OS was 55.8% in the non-pCR group compared with 80.0% in pCR (*p* = 0.0007). The median survival was 69.6 months (95% CI = 59.21–95.34) in the non-pCR group [42]. While the proportion of non-pCR has decreased with the advent of chemotherapy + immunotherapy combination, it can still reach 60%. However, their survival remains inferior to those with pCR, regardless of the chemotherapy and immunotherapy combination, with a median survival unreached for pCR patients vs. 26.6 months for non-pCR patients in the chemotherapy + Nivolumab arm [43]. Nonetheless, the incorporation of immunotherapy within this population confers a benefit compared to non-pCR patients receiving chemotherapy alone, with a 31% reduction in the risk of relapse (HR = 0.69, 95%CI, 0.55–0.85) in the KEYNOTE 671 study, and this benefit is consistent across various studies. Although immunotherapy improves the survival of this population, it is imperative to design clinical trials explicitly targeting this patient group to optimize their clinical outcomes. A promising approach is to swiftly explore new therapeutic targets to ensure effective treatment maintenance. New therapeutic options such as targeted chemotherapy, antibody-drug conjugates (ADCs), and immune checkpoint inhibitors (such as anti-TIGIT) hold considerable potential in this context. Whether used as monotherapy or in combination with anti-PD1 immunotherapy, these strategies could offer new avenues to improve survival and quality of life for non-pCR NSCLC patients.

### 3.2. Stage II

Stage II non-small cell lung cancer (NSCLC) patients present with distinct prognostic characteristics compared to those diagnosed with stage III disease. Specifically, for stage IIA, the median survival time was not reached, while it was 66 months for stage IIB. In contrast, median survival times were 29 months for stage IIIA and 19 months for stage IIIB.

The standard treatment for this population remained primary surgery. Thus, in the era of neoadjuvant chemotherapy-immunotherapy, a question arises for this population: The risk of overtreatment, with the risk of toxicity and the non-realization of surgical treatment. In these studies, fewer than 30% of patients presented with stage II. And within this population, there was no significant benefit in relapse-free survival (HR = 0.87 (0.48–1.56)). Furthermore, close to 15% of surgical treatments were canceled [34,43]. For this population, the role of adjuvant treatment is optional and does not delay surgical intervention.

The IMpower 010 trial included patients with completely resected stage IB to IIIA NSCLC. Following the completion of mandatory adjuvant platinum-doublet chemotherapy (of at least one cycle), patients were randomly assigned to receive either atezolizumab 1200 mg every 3 weeks for up to 1 year or best supportive care [12]. An improvement in disease-free survival (DFS) was observed in the atezolizumab group (median not reached vs. 35.3 months with best supportive care; HR 0.66, 95% CI 0.5–0.88; *p* = 0.004) in patients with stage II–IIIA disease and PD-L1 expression ≥ 1%, with an even greater benefit observed in patients with PD-L1 expression ≥ 50% [12].

The PEARLS trial investigated adjuvant pembrolizumab vs. placebo for patients with stage II–IIIA, completely resected early-stage NSCLC. DFS was significantly improved with pembrolizumab in the overall population (median 53.6 vs 42.0 months; HR 0.76; 95% CI 0.63–0.91; *p* = 0.0014); however, the significance boundary was not crossed for the population with tumor proportion score (TPS) ≥ 50% [44].

Our recommendation would be to prioritize upfront surgery followed by adjuvant chemotherapy and then immunotherapy for this population.

### 3.3. Oncogene-Driven NSCLC Population

Patients with advanced-stage NSCLC driven by oncogenes typically show limited benefits from single-agent immune checkpoint inhibitors. Therefore, targeted therapies are often prioritized over ICIs or chemoimmunotherapy in these cases [45]. Patients with EGFR or ALK mutations were excluded to clinical trials in neoadjuvant setting. Whether findings from advanced-stage oncogene-driven NSCLC can be applied to early-stage disease remains uncertain.

For instance, in trials such as IMpower 010, patients with tumors harboring EGFR and/or ALK alterations didn’t experience a disease-free survival (DFS) benefit from adjuvant atezolizumab, while never-smokers and those with EGFR mutations saw DFS benefits in trials like PEARLS [12,44]. Notably, in patients with resected stage II–IIIA NSCLC and EGFR mutations, adjuvant treatment with osimertinib significantly improved DFS and OS [46]. This supports the prioritization of TKIs over neoadjuvant ICIs in patients with resected EGFR-mutant NSCLC, making osimertinib an attractive postoperative therapeutic option.

Several ongoing trials are investigating neoadjuvant and adjuvant TKIs, some in combination with chemotherapy or ICIs in the neoadjuvant setting. These trials aim to determine the optimal strategy for patients with oncogene-addicted early-stage NSCLC. Currently, when considering neoadjuvant ICI treatment for NSCLCs with oncogenic alterations beyond EGFR and ALK, clinicians should openly discuss the best available evidence with patients and select an individualized approach [45].

## 4. Conclusions

The combination of neoadjuvant immunotherapy and chemotherapy has clearly become a standard in the management of localized NSCLC, providing significant benefits in terms of progression-free survival (EFS) and OS. However, critical questions persist, notably regarding patient selection criteria and subsequent management strategies. For example, it is essential to ensure the maintenance of patients’ quality of life during and after treatment while also limiting associated toxicities.

We therefore wish to begin by recalling, surgery remains the cornerstone of localized NSCLC management, so it’s crucial during tumor board meetings to assess the resectability of the patient independently of neoadjuvant treatment and potential response. As we’ve emphasized, a significant percentage of patients may not be eligible for surgery after neoadjuvant treatment due to disease progression or toxicity. Therefore, in our view, a stage II patient should undergo primary surgery followed by adjuvant treatment and immunotherapy. For stage III patients whose resectability is uncertain at diagnosis, they should receive chemotherapy combined with radiotherapy followed by immunotherapy, in accordance with recommendations and the proven benefit demonstrated by the PACIFIC study [47].

Furthermore, despite achieving a significant rate of complete pathological response (pCR), a substantial proportion of patients fail to reach this goal, with rates ranging from 20% to 40% across different clinical trials. This underscores the need to develop effective strategies to address non-responders and optimize therapeutic outcomes. For this population, it would be important to develop adjuvant trials evaluating the contribution of other molecules, such as conjugated antibodies, bispecific antibodies, more or less associated with immunotherapy.

Moreover, the identification of predictive biomarkers for patient stratification remains a major challenge, especially concerning the selection of candidates for adjuvant immunotherapy or maintenance therapy after neoadjuvant treatment. ctDNA appears to be a promising biomarker. Indeed, patients who do not present or have negative ctDNA post-operatively after neoadjuvant chemotherapy and immunotherapy followed by surgery could be monitored and deferred from adjuvant immunotherapy, thereby limiting toxicity. Patients with persistent positive ctDNA, on the other hand, could receive adjuvant immunotherapy or even treatment intensification.

Thus, while the integration of chemotherapy and immunotherapy holds great promise for enhancing therapeutic responses in localized NSCLC, further research is warranted to elucidate optimal patient selection criteria and refine therapeutic approaches to maximize clinical benefits.

## Figures and Tables

**Table 1:** Summary of histologic response and survival outcomes in patients treated with chemoimmunotherapy in originator studies. * Not reached; ---Not recorded.

	CM816	AEGEAN	KN671	CM77T
**Total number of study patients**	358	802	797	461
**Immunotherapy used**	Nivolumab	Durvalumab	Pembrolizumab	Nivolumab
**Patients operated** in the experimental arm (%)	83.2%	77.6%	82.1%	78%
**R0 (%)** in the experimental arm	83.2%	92%	94.7%	89%
**pCR (%)** in the experimental arm	24%	17.2%	18.1%	25.3%
**MPR (%)** In the experimental arm	36.9%	33.3%	30.2%	35.4%
**Median of PFS (month)** In the experimental arm	31.6	NR *	NR *	NR *
**Estimated OS** In the experimental arm	NR * At 36 month	---	80.9% At 24 month	---

**Table 2:** Type of surgery after neoadjuvant chemotherapy and immunotherapy and safety.

	CM816		AEGEAN		KN671		CM77T	
	Nivolumab	Chemo	Durva	Placebo	Pembro	Placebo	Nivolumab	Placebo
Safety & Surgery								
Patients with definitive surgery	149 (83.2)	135 (75.4)	324 (81.0)	327 (81.3)	326 (82.1)	318 (79.5)	178 (78)	178 (77)
Type of surgery: - Lobectomy - Pneumonectomy	115 (77.2) 25 (16.8)	82 (60.7) 34 (25.2)	238 (65.0) 27 (7.4)	221 (59.1) 29 (7.8)	256 (78.8) 37 (11.4)	238 (75.1) 39 (12.3)	142 (80) 16 (9)	128 (72) 24 (14)
Patients with cancelled surgery: - Disease progression - Adverse Event	28 (15,6) 12 (6.7) 2 (1,1)	37 (20,7) 17 (9.5) 1 (0.6)	76 (19.0) 27 (6.8) 7 (1.8)	75 (18.7) 30 (7.5) 5 (1.2)	71 (17.9) 15 (3.8) 25 (6.3)	82 (20.5) 26 (6.5) 17 (4.2)	46 (20) 13 (6) 7 (3)	50 (22) 22 (10) 4 (2)
**Adverses event identified as surgical complication**								
All	41.6%	46.7%	---	---	---	---	73 (41)	69 (39)
Grade 3 and 4	11.4%	14.8%	---	---	---	---	21 (12)	21 (12)
**Treatment related adverses event Grade 3 & 4**								
All	59 (33.5)	65 (36.9)	129 (32.4)	131 (32.9)	(44.9)	(37.3)	61 (27)	52 (23)
Leading to discontinuation of treatment	10 (5.7)	6 (3.4)	26 (6.7)	15 (3.8)	(12.6)	(5.3)	18 (8)	9 (4)
Serious	15 (8.5)	14 (8.0)	32,4%	32,9%	(17.7)	(14.3)	---	---
Death	0	3 (1.7)	7 (1.7)	2 (0.5)	1 (0.002)	1 (0.002)	2 (1)	0
